# Adaptation and validation of social accountability measures in the context of contraceptive services in Ghana and Tanzania

**DOI:** 10.1186/s12939-020-01286-1

**Published:** 2020-10-15

**Authors:** Victoria Boydell, Petrus S. Steyn, Joanna Paula Cordero, Ndema Habib, My Huong Nguyen, Dela Nai, Donat Shamba

**Affiliations:** 1 Global Health Institute, Geneva Graduate Institute, Geneva, Switzerland; 2 UNDP/UNFPA/UNICEF/WHO/World Bank Special Programme of Research, Development and Research Training in Human Reproduction (HRP Research), World Health Organization, Geneva, Switzerland; 3 Population Council, Accra, Ghana; 4 Department of Health Systems, Impact Evaluation and Policy, Ifakara Health Institute, Dar es Salaam, Tanzania

**Keywords:** Social accountability, Measurement, Contraception, Tanzania, Ghana

## Abstract

**Background:**

Changes in the values, attitudes, and interactions of both service users and health care providers are central to social accountability processes in reproductive health. However, there is little consensus on how best to measure these latent changes. This paper reports on the adaptation and validation of measures that capture these changes in Tanzania and Ghana.

**Methods:**

The CaPSAI theory of change determined the dimensions of the measure, and we adapted existing items for the survey items. Trained data collectors used a survey to collect data from 752 women in Tanzania and 750 women in Ghana attending contraceptive services. We used reliability analysis, exploratory, and confirmatory factor analysis to assess the validity and reliability of these measures in each country.

**Results:**

The measure has high construct validity and reliability in both countries. We identified several subscales in both countries, 10 subscales in Tanzania, and 11 subscales in Ghana. Many of the domains and items were shared across both settings.

**Conclusion:**

The study suggests that the multi-dimensional scales have high construct validity and reliability in both countries. Though there were differences in the two country contexts and in items and scales, there was convergence in the analysis that suggests that this measure may be relevant in different settings and should be validated in new settings.

**Trial registration:**

ACTRN12619000378123.

## Background

Considerable progress has been made in increasing women’s access to modern contraception [35]. Nevertheless, there is continuing unmet need, particularly for those with less education, lower incomes, and younger age, and high rates of discontinuation due to poor quality of care and negative patient experience [10, 35, 39]. In recent years, there have been renewed efforts to understand better and address issues surrounding the quality of care and clients’ experiences of contraceptive services. Studies have identified a range of quality care issues that negatively affect clients’ experiences of interacting with contraceptive services [3, 17, 25].

Community engagement and more patient-centred care have come to the forefront as essential mechanisms to address issues around quality of care and poor client experience [24, 40]. Evidence increasingly indicates the positive effects of social accountability on improving the quality of care and experience in other health sectors and concerning contraception specifically [5, 13, 38]. We define social accountability as “citizens’ efforts at ongoing meaningful collective engagement with public institutions for accountability in the provision of public goods.” ([18], pg 161). It is often best recognized through the tools used to facilitate the process, such as community scorecards, social audits, and participatory budget. As a potentially transformative change process, social accountability transforms the norms, values, and attitudes of those seeking services and those providing them, which together can bring about changes in the health system delivery and, in the longer term, population health outcomes. However, the all-important changes in service users and providers’ norms, values, and attitudes are often not measured, possibly because they are not observable but rather latent and multi-dimensional.

Though few validated measures aim to capture these variables, a notable exception is CARE’s Women’s Voice tool that provides an important starting point for researchers in this area [34]. As part of a more extensive complex intervention study [36], we adapted and validated measures of service users’ attitudes and behaviors in a social accountability process to improve family planning services. In this paper, we describe the process of adapting the measures and assessing their validity and reliability in Ghana and Tanzania.

## Methods

The larger study’s theory of change about how service users’ attitudes changed during the social accountability process determined the dimensions of the measures. To develop the survey items, we adapted existing items. Trained data collectors used a survey to collect data from women attending contraceptive services in Tanzania and Ghana. We used reliability analysis, exploratory, and confirmatory factor analysis to assess the validity and reliability of these measures in each country.

The development of these measures is part of a more extensive complex intervention study, Community and Provider Social Accountability Intervention (CaPSAI), undertaken in Tanzania and Ghana [36]. These countries were selected based on the following criteria: (1) existence of a national civil society organization (CSO) partner with experience in social accountability, (2) low modern contraceptive prevalence rate, (3) availability of contraceptive services at the point of contact where cost is not a barrier to access, (4) an enabling environment in terms of the potential for the health system to respond to the social accountability, and (5) the existence of formal structures linking the community with the health system (e.g. health committees).

The study took place in Mbeya City and Chunya districts in Tanzania, and Abura-Asebu-Kwamankese, Komenda-Edna-Eguafo-Abirem, and Mfantsiman districts in Ghana. The sites were selected based on (1) the provision of contraceptive services; (2) availability of the following methods: a barrier method, a short and long-acting method, emergency contraception, and at least referral for permanent methods in districts; and (3) no social accountability interventions in FP/C currently underway [36] Table 1

**Table 1:** Characteristics of the study settings [36]

Type of facilities	Ghana (***n*** = 8)	Tanzania (***n*** = 8)
District Hospital	1	0
Health Centre /Clinic	6	2
Health Post (Community-based Health Planning Services - Ghana)	1	Not Applicable
Dispensary (Tanzania)	Not Applicable	6

.

### Dimensions of the measure

**Fig. 1:**
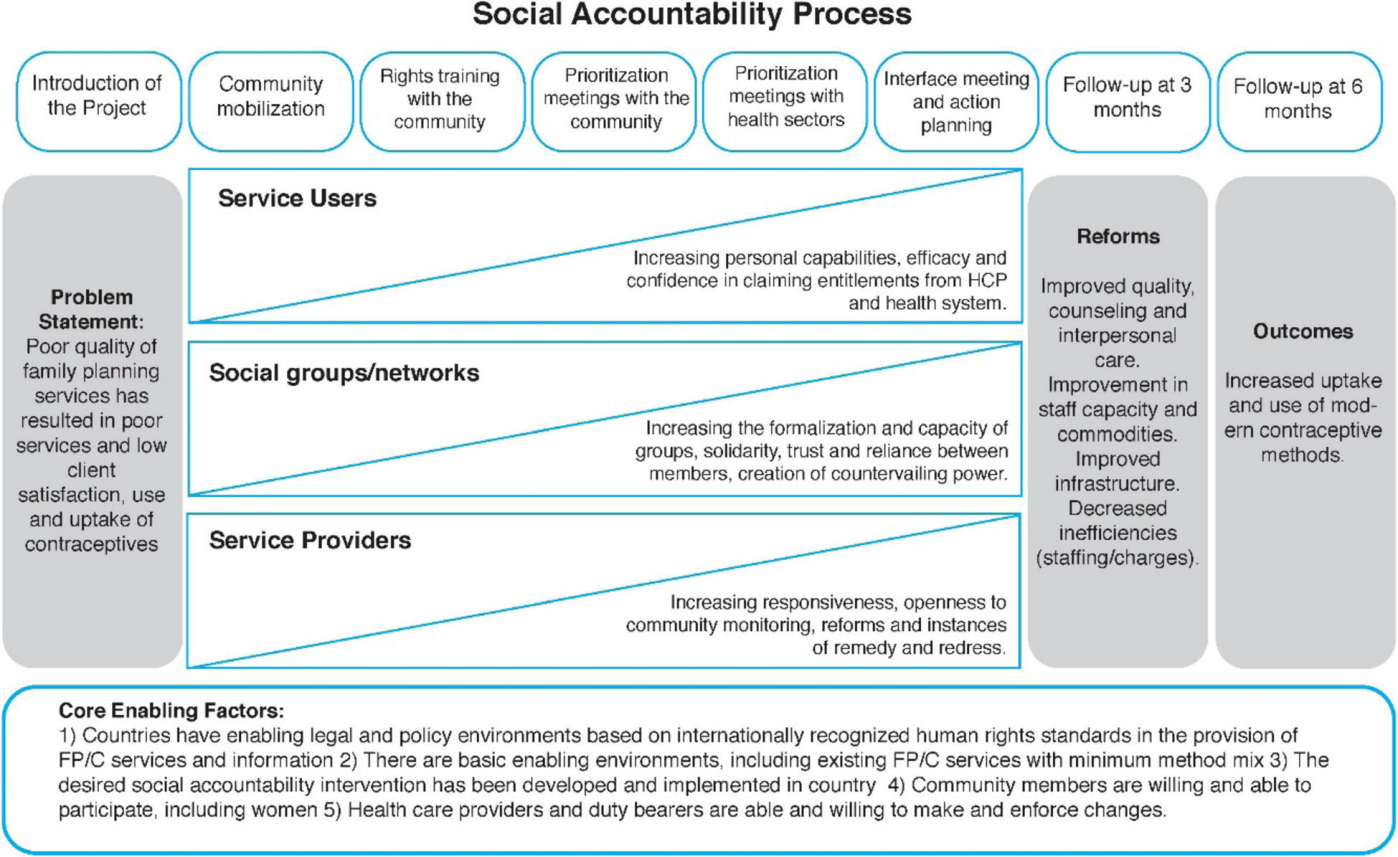
CaPSAI Theory of Change

The theory of change drew on existing empirical and theoretical work on social accountability more broadly and specifically related to sexual and reproductive health (see [7, 8, 36]). This informed the dimensions in the measure. The theory of change, Fig. 1, details the inputs of the social accountability process (across the top of the diagram), how these correspond to the cumulative intermediate outcomes at three levels: (1) service users, (2) social networks and (3) service providers, which in turn, effect intended reforms in the quality of care that contribute to contraceptive choice, including increased uptake and use of modern contraceptive methods. As detailed in elsewhere, social accountability engages community members and health services actors in dialogues to identify shared challenges and develop action plans that can lead to improvements in service quality in the health system and in at the individual level, the service user or potential user knowledge and engagement with the health system, both in terms of their own health seeking behaviour and their participation in dialogues with authorities [36].

### Item adaptation and development

Following the development of the dimensions, we identified existing validated measures for each domain. We drew heavily on CARE’s Women VOICES tool, a validated measure the aimed to capture similar intermediate outcomes concerning maternal health in Malawi [34]. We added three domains to those used in the VOICES tool to represent the CaPSAI theory of change fully. First was the ‘Knowledge and awareness of rights’ domain that aimed to capture the service user’s perception of rights were also included [42]. For service users’ efficacy with health care providers, we used the National Health Service (NHS) measure of patient activation [28]. To capture changes in service user’s awareness of how to bring about changes and improve their local services, we created items based on theoretical work on political capabilities [22, 41]. We adapted five VOICES validated scales with acceptable reliability to the contraceptive services and local context; for example, family planning services have a charge in Ghana but not in Tanzania. A total of 14 domains were included with 75 items (see Tables 2 and 3). A five-point Likert scale was used for all the item with the exception of two sets of items had different ranges in their original format. A 6 point scale was used for Self-efficacy with health care providers (set A) ‘Women’s participation in household decision- making’ and a dichotomous scale was used for ‘Self-efficacy with health care providers (set A)’ as originally used [28, 34].

**Table 2:** Domains identified based on Theory of Change

Domains	Number of Items
Knowledge and awareness of rights ^c^	8^b^
Women’s participation in household decision- making^a^	10^b^
Self-efficacy with health care providers ^c^	13^b^
Self-efficacy for participation at community meetings^a^	6 ^b^
Perception of service quality ^a^	6^b^
Political capabilities^c^	5^b^
Collective efficacy ^a^	4^b^
Community support in time of crisis ^a^	4^b^
Mutual responsibility for and support of services^a^	5^b^
Participation in negotiated spaces ^a^	4
Joint monitoring and accountability of services ^a^	2
Transparency ^a^	3
Equity of negotiated spaces ^a^	3
Quality of negotiated spaces ^a^	3

^a^validated from CARE’s Women VOICES Tool

^b^scales tested in this study

^c^new scales

**Table 3:** List of items included, per domain

Domain	Likert Range	No.	Item
Knowledge and awareness of rights	1–5	38	A healthcare provider can refuse to provide me family planning services because of who I am.
		39	The government ensures that family planning services are free of cost (Tanzania) or The government ensures that family planning methods are free of cost (Ghana)
		40	I have the right to privacy during my family planning visit.^a^
		41	The healthcare provider should not share my information with other people.
		42	If I am unhappy with the care I received, I know there are ways to make a complaint.^a^
		43	Healthcare providers must answer all my health-related questions.^a^
		44	Healthcare providers should inform me about the different family planning options^a^
		45	I can refuse any family planning method offered if I do not want to use it.^a^
Women’s participation in household decision- making	1–6	46	First, would you tell me which member of your household usually makes decisions about your health care? ^a^
		47	Which member of your household usually makes decisions about making large household purchases? ^a^
		48	Which member of your household usually makes decisions about making household purchases for daily needs?^a^
		49	Which member of your household usually makes decisions about when you will visit family/relatives/friends?^a^
		50	Which member of your household usually makes decisions about when your whole household will visit family/relatives/friends?^a^
		51	Which member of your household usually makes decisions about how to use the money that you bring into the household?^a^
		52	Which member of your household usually makes decisions about how to use the money your husband/partner brings into the household?^a^
		53	Which member of your household usually makes decisions about whether you or you and your husband/partner use family planning?^a^
		54	Which member of your household usually makes decisions about where you will receive family planning?^a^
		55	Which member of your household usually makes decisions about if you will be tested for the AIDS virus? ^a^
		56	Which member of your household usually makes decisions about how many children you will have? ^a^
Self-efficacy with health care providers A	1–2	57	After your consultation with the health care provider today, do you know what your reproductive and family planning options are? ^a^
		58	After your consultation with the health care provider today, do you feel that you can act on your choice for family planning? ^a^
		59	Do you know what help you need to make a decision? ^a^
Self-efficacy with health care providers B	1–5	65	I felt like I could discuss my problems, question and concerns with the health care provider without feeling embarrassed.
		66	One of the providers or staff refused to offer me the service I wanted to receive. (reverse-coded)^a^
		67	The provider ignored my request or my preferences today.^a^
		68	I felt like the provider did not listen to what I was saying (reverse-coded)^a^
		69	A provider strongly encouraged me to use one family planning that was different to the one I wanted (reverse-coded)^a^
		70	I have the right to choose my family planning method.
Self-efficacy for participation at community meetings	1–5	71	How sure are you that you could attend a community meeting if your family did not support you to participate?^a^
		72	How sure are you that you could attend a community meeting if your family said you could not go? ^a^
		73	How sure are you that you could attend a community meeting if your family would not help with your household duties so that you could attend? ^a^
		74	How sure are you that you could express your opinion at a community meeting? ^a^
		75	How sure are you that you could express your opinion at a community meeting if a few people did not agree with what you were saying? ^a^
		76	How sure are you that you could express your opinion at a community meeting if many people did not agree with what you were saying? ^a^
Perception of service quality	1–5	77	The staff at this health facility have high quality family planning services ^a^
		78	The staff at this health facility ensures privacy and confidentiality when providing services. ^a^
		79	The health facility is clean. ^a^
		80	At this health facility, if women choose, they can bring their husband/partner for the family planning consultation. ^a^
		81	At this health facility, if women choose, they can bring a family member or friend for the family planning consultation. ^a^
		82	Unmarried women can access family planning and reproductive health service at the health facility.
Political capabilities	1–5	83	Today, if I went to the clinic, I believe I could get family planning I wanted without facing any barriers of cost, age and marital status.
		84	Anyone outside of the clinic, like friends or community members, can help you access your right to quality family planning services.
		85	Health providers and district government officials can directly influence the quality of your local family planning services? ^a^
		86	Challenging people of influence is the best way to change family planning services in the clinic. ^a^
		87	Collaboration with people of influence is the best way to change family planning services in the clinic. ^a^
Collective efficacy	1–5	88	How sure are you that the people in your community could work together to improve family planning services in this community? ^a^
		89	How sure are you that the people in your community could work together to improve how women are treated at the health facility? ^a^
		90	How sure are you that the people in your community could work together to obtain government services and entitlements? ^a^
		91	How sure are you that the people in your community could work together to improve the health and well- being of women in this community? ^a^
Community support in time of crisis	1–5	92	How sure are you that there is someone in your community, apart from your immediate family, who you could go to for advice? ^a^
		93	How sure are you that there is someone in your community, apart from your immediate family, who could take you to the clinic? ^a^
		94	How sure are you that there is someone in your community, apart from your immediate family, who would help care for your children or household while you are away? ^a^
		95	How sure are you that there is someone in your community, apart from your immediate family, who would loan you money for transport? ^a^
Mutual responsibility for and support of services	1–5	99	Who could have the most impact on making sure that women are treated with respect by health workers? ^a^
		100	Who could have the most impact on making sure that women have transportation to the hospital for permanent methods of contraception? ^a^
		101	Who could have the most impact on increasing the number of days a health worker visits your community? ^a^
		102	Who could have the most impact on making sure the poorest and most vulnerable women in the community receive care? ^a^
		103	Who could have the most impact on getting funding to improve health services in this community? ^a^

(^a^) retained from [34]

We ascertained content validity of the overall items through consultation with experts in social accountability and family planning, and the Principal Investigators who reviewed the questionnaire. Also, the World Health Organization (WHO) Forms Committee, which was composed of technical experts in contraception, social scientists, biostatisticians, and data managers, reviewed the instruments.

### Survey administration

English surveys were available in both countries and translated in Akan in Ghana and into Kiswahili in Tanzania. Back translation was within a normal range, and pretesting the questionnaire was satisfactory for use in the study populations.

The same eligibility criteria for participants were used in both sites (see [36]). A sample of over 750 women aged 15 to 49 years accessing contraceptive services was interviewed prior to the start of the intervention in each country. Sampling was calculated using a priori sample size calculation with the ratio of 10 responses per item ratio and guidance of more than 500, which equals a very good sample for validation [4, 34]. Our sample calculation was based on 75 items of the full survey of items, including post test items. The same items were administered as part of a client exit interview survey in Tanzania and Ghana. The item related to cost of service varied between the countries as there is a nominal fee charged in Ghana. In Tanzania, the item was ‘The government ensures that family planning services are free of cost’ and in Ghana it was ‘The government ensures that family planning methods are free of cost.’

A total of 118 questions were asked of respondents upon leaving a facility, and only 58 scale items and 9 domains were included in the following analysis. Five domains and 17 items were excluded from this analysis because they were not scales or were items that applied after the intervention had been implemented.

The other survey items included questions about demographics, reproductive and family planning history, relationship status, income, occupation, and religion.

Client exit interviews were conducted on the day of recruitment at the facility in a private location. In Ghana, a total of 15 data collectors (5 females and 10 males) were trained in the survey over 3-day training in April 2018. In Tanzania, a total of 14 data collectors (8 females and 6 males) were trained over in the survey over 5 days in March 2018. Data collection was conducted using a tablet-based questionnaire to capture real-time data using OpenClinica and was later uploaded onto a secure server. Data collection in Tanzania started on 26 March and was completed on 25 May 2018, and all respondents choose to be interviewed in Kiswahili. In Ghana, data collection started on 9 April 2018 and was completed on 4 June 2018, and 46.4% choose to be interviewed in English while 53.6% choose to be interviewed in Akan.

Having being assessed for eligibility, respondents completed the informed consent process. There were no incentives given to women and girls to participate in the study. However, study participants who agreed to participate were reimbursed for their travel cost, where it was permitted by country-specific ethical requirements. In Ghana, the research team supported the travel cost to the facility with five Ghana cedis (~ 1 US dollar) given after the interview. In Tanzania, no reimbursements were given.

### Psychometric analysis

We assessed the item and scale reliability followed by exploratory factor analysis (EFA) and confirmatory factor analysis (CFA) for each country. We assessed the reliability of items and scales to test the internal consistency. The EFA aimed to identify the relationships among items and then group the items as part of a factor. CFA was conducted to confirm the theory behind the grouping of items.

We started with assessing reliability using the Cronbach’s alpha to determine item -to- item correlation (or homogeneity) of all 58 observed items and determined the overall alpha for each scale in each country. A Cronbach’s alpha of 0.60 was considered acceptable reliability and 0.70 or higher to be good reliability [16]. Items were removed, according to standard procedures, if the overall alpha improved substantially if an item was removed [23]. Scales with Cronbach’s alpha of ≥.60 were retained [34].

We conducted exploratory factor analysis (EFA) to determine how all 58 observed items clustered together and explore the underlying factor structure in each country. We computed the communality for each item, defined as the proportion of variance in the item attributable to common factors; and used a Kaiser-Meyer-Olkin (KMO) of Sampling Adequacy to assess the suitability of items for the factor analysis. Overall, and factors with KMO value > 0.5, for factor analysis were considered suitable for factor analysis [19, 21]. To determine the factors, we used eigenvalues in accordance with the Kaiser Criterion [20]. We examined the eigenvalues and the scree plot of eigenvalues, and factors with Eigenvalues greater than 1.0 were retained [20].

We used a rotated factor analysis using the maximum likelihood estimation (MLE) with oblique oblimin rotation to determine the factor loadings and variance. Factor loadings assess how items are weighted for each factor and the correlation between the variables and the factor. We used the proportion of variance in the item explained by the factors jointly to assess the reliability of the item in the context of all the factors. Items with factor loadings with values less than 0.40 were excluded. A minimum of three items per factor is recommended, and factors with two items or less were excluded [26].

Confirmatory Factor Analysis (CFA) was done to confirm whether the constructs identified in EFA had a good fit statistically. We applied three recommended models to test for goodness-of-fit [32]. The Standardized Root Mean Square Residual (SRMR) is a measure of the mean absolute correlation residual with a threshold of ≤0.08; the Root Mean Square Error of Approximation (RMSEA) measures the estimated discrepancy between the population and model-implied population covariance matrices per degree of freedom, and a score of ≤0.06 is acceptable, and Comparative Fit Index (CFI) measures the relative improvement in the fit of a researcher’s model over that of a baseline model, and a CFI ≥ 0.95 considered an acceptable fit [6]. The CFA structural model was is presented for each country.

## Results

### Demographic characteristics

In total, 750 in Ghana, and 752 women in Tanzania completed the survey. This sample is based on 10 respondents per item to produce a reliable estimate [6]. Table 4 shows the demographic characteristics of respondents.

**Table 4:** Demographic Characteristics of Ghana and Tanzania Sample

	Ghana n (%)	Tanzania n (%)
**Age, years**		
Mean (SD) [Min, Max]	28.4 (7.1) [15, 49]	27.8 (6.3) [16., 47]
Median (IR)	27 (23, 33)	27 (23, 32)
≤ 20	94 (12.5)	83 (11.1)
20–39	636 (70.1)	567 (75.6)
> 39	130 (17.4)	100 (13.3)
**Marital status**		
Currently married	476 (63.5)	636 (84.8)
Never married	225 (30.0)	77 (10.3)
Other *(Cohabitation, Fiance, no husband, separated, divorced)*	49 (6.5)	37 (4.9)
**Methods currently using (among those using)**		
Female sterilization	7 (1.1%)	11 (1.5%)
Male sterilization	2 (0.3)	3 (0.4)
IUD	8 (1.3)	40 (5.5)
Injectables	456 (71.7)	391 (53.7)
Implants	128 (20.1)	217 (29.8)
Pill	32 (5.0)	122 (16.8)
Male condom	17 (2.7)	53 (7.3)
Female condom	5 (0.8)	9 (1.2)
Emergency contraception	11 (1.7)	1 (0.1)
Standard days method	7 (1.1)	24 (3.3)
Lactational amenorrhea method	6 (0.9)	27 (3.7)
Other (Rhythm method/ withdrawal)	22 (3.4)	73 (10.1)
**Highest level of school completed**		
No formal schooling	131 (17.5)	29 (3.9)
Some primary school	243 (32.4)	48 (6.4)
Completed primary school	194 (25.9)	423 (56.4)
Some secondary school (some and completed)	109 (14.5)	222 (29.6)
Any tertiary education	73 (9.7)	28 (3.7)
**Reading level**		
Cannot read at all	303 (40.4)	34 (4.5)
Able to read only part of the sentence	187 (24.9)	105 (14.0)
Able to read whole sentence	259 (34.5)	611 (81.5)

The mean age of the women that participated in the survey was 28.4 in Ghana and 27.8 in Tanzania. In Tanzania, 84.8% of women were currently married compared to 63.5% in Ghana. A higher percentage of women in Tanzania were never-married (30.0%) than Ghana (10.3%). In both countries, injectable and implants were the predominant current contraceptive method.

### Analysis

To assess the scales in Tanzania and Ghana, we conducted three sets of analyses to (1) assess the reliability of the subscales, (2) determine how many factors to retain and reduce the items, and (3) verify our proposed groupings separately per country.

### Tanzania

We started with a reliability analysis using the Cronbach’s alpha to determine item -to -item correlation of all 58 observed items and determine the overall alpha for each subscales in Tanzania (see Table 5). The standardized alpha was greater than 0.60 (the acceptable reliability in social science research) for 6 of the 11 subscales and was retained. When the alpha was lesser than 0.70, items were removed if their removal improved the overall alpha scale. A total of 6 items were removed; this included questions 38, 65, 70, 82, 83, and 84. Subscales with Cronbach’s alpha of ≥.60 were retained.

**Table 5:** Reliability Analysis in Tanzania

**Qn #**	Scale mean if item deleted	Scale Standard Deviation	Variance	Item total Correlation with Total	Alpha if item deleted
Knowledge and Awareness of Rights					
38*	2.71	1.42	2.01	−0.17	**0.83**
39	1.46	0.61	0.37	0.55	0.72
40	1.61	0.65	0.42	0.58	0.71
41	1.63	0.70	0.49	0.55	0.72
42	2.24	1.26	1.58	0.47	0.73
43	1.64	0.60	0.36	0.62	0.70
44	1.62	0.59	0.34	0.67	0.69
45	1.78	1.02	1.04	0.49	0.73
**Reliability Coeffcients**		Alpha = 0.83		N of cases 752	N of items: 7
Women’s participation in household decision-making (all items)					
46†	1.60	0.49	0.24	0.51	**0.88**
47†	1.41	0.49	0.24	0.48	0.88
48†	1.69	0.46	0.21	0.56	0.88
49†	1.64	0.48	0.23	0.66	0.87
50†	1.62	0.48	0.23	0.65	0.87
51†	1.72	0.45	0.20	0.67	0.87
52†	1.63	0.48	0.23	0.51	0.88
53†	1.81	0.39	0.15	0.68	0.87
54†	1.84	0.36	0.13	0.69	0.87
55†	1.82	0.38	0.15	0.62	0.87
56†	1.76	0.43	0.18	0.56	0.88
**Reliability Coeffcients**		Alpha = 0.83		N of cases 752	N of items: 10
Self efficacy with health care providers (Qn 57, 58 & 59)					
57**	0.94	0.23	0.05	0.43	0.43
58**	0.95	0.22	0.05	0.51	0.31
59**	0.76	0.43	0.18	0.26	0.68
**Reliability Coeffcients**		Alpha = 0.59		N of cases 752	N of items: 2
Self efficacy with health care providers (all items)					
65	2.67	1.38	1.89	−0.16	**0.74**
66*	1.95	0.90	0.81	0.55	0.46
67*	1.82	0.81	0.66	0.58	0.45
68*	1.89	0.86	0.74	0.53	0.47
69*	2.03	1.02	1.04	0.48	0.50
70	1.55	0.73	0.54	0.18	**0.62**
**Reliability Coeffcients**		Alpha = 0.81		N of cases 752	N of items: 3
Self-efficacy for participation at community meetings (all items) (Qn 71–73)					
71	1.88	1.27	1.61	0.73	0.75
72	2.05	1.31	1.72	0.80	0.69
73	2.06	1.26	1.59	0.60	**0.88**
**Reliability Coeffcients**		Alpha = 0.88		N of cases 752	N of items:2
Self-efficacy for participation at community meetings (all items)					
74	2.02	1.30	1.69	0.65	0.93
75	2.49	1.42	2.02	0.85	0.76
76	2.63	1.49	2.22	0.81	0.78
**Reliability Coeffcients**		Alpha = 0.88		N of cases 752	N of items: 2
Perception of service quality (all items)					
77	1.62	0.64	0.41	0.58	0.64
78	1.59	0.59	0.35	0.59	0.64
79	1.70	0.65	0.42	0.53	0.66
80	1.70	0.73	0.53	0.49	0.67
81	2.26	1.08	1.17	0.35	0.71
82	2.18	1.11	1.23	0.21	**0.75**
**Reliability Coeffcients**		Alpha = 0.75		N of cases 752	N of items: 5
Political Capabilities					
83	1.99	1.10	1.21	0.06	**0.79**
84	2.60	1.25	1.57	0.44	**0.65**
85	1.93	0.85	0.72	0.52	0.61
86	2.10	1.03	1.06	0.66	0.55
87	2.09	1.03	1.05	0.65	0.55
**Reliability Coeffcients**		Alpha = 0.81		N of cases 752	N of items:4
**Collective efficacy (all items)**					
88	1.91	1.23	1.51	0.67	0.85
89	1.91	1.13	1.28	0.79	0.80
90	1.80	1.06	1.12	0.71	0.83
91	1.86	1.10	1.21	0.70	0.84
**Reliability Coeffcients**		Alpha = 0.87		N of cases 0751	N of items:4
Community support in time of crisis (all items)					
92	1.72	1.27	1.61	0.39	0.57
93	1.70	1.13	1.28	0.49	0.50
94	1.90	1.28	1.64	0.39	0.57
95	2.11	1.38	1.90	0.37	0.59
**Reliability Coeffcients**		Alpha = 0.63		N of cases 752	N of items:3
Mutual responsibility for and support of services (all items)					
99¥	1.69	0.50	0.25	0.33	0.51
100¥	1.39	0.64	0.41	0.32	0.52
101¥	1.36	0.55	0.30	0.44	0.45
102¥	1.51	0.53	0.28	0.39	0.48
103¥	1.27	0.47	0.22	0.19	0.59
**Reliability Coeffcients**		Alpha = 0.60		N of cases 752	N of items:4

* Reverse coded

** Yes/No Response items

† 2 Response items

¥ 3 Response items

We undertook a KMO measure of sampling adequacy, and all items had a KMO of 0.5. The overall KMO score was 0.85, suggesting that there is a sufficient correlation between the variables to conduct exploratory factor analysis.

The EFA used principal factors with oblique oblimin rotation for all 58 items that yielded 11 factors. All factors had eigenvalues greater than 1.0; see Table 6 (which explained 100% of the variance).

**Table 6:** Eigenvalues in Tanzania

Factor	Eigenvalue
**1**	23.23
**2**	20.77
**3**	15.40
**4**	8.16
**5**	6.97
**6**	4.35
**7**	3.96
**8**	2.99
**9**	2.38
**10**	1.87
**11**	1.69

In total, five items loaded on factor 1, five items loaded on factor 2, four items loaded on to factor 3, four items loaded on to factor 4, two items loaded on to factor 5, three items loaded onto factor 6, three items loaded onto factor 7, three items loaded onto factor 8, three items loaded onto factor 9, three items loaded onto factor 10, and two items loaded onto factor 11 Table 7. We discarded items with factor loadings of less than 0.4, and 12 items were removed, see Table 7. Factors with less than two items were excluded as this is not a sufficient number for factor analysis. On this basis, two factors were removed, and 10 factors were retained. A total of 40 items were retained.

**Table 7:** Factor loadings for Tanzania

XSU QN #	FACTOR 1	FACTOR 2	FACTOR 3	FACTOR 4	FACTOR 5	FACTOR 6	FACTOR 7	FACTOR 8	FACTOR 9	FACTOR 10	FACTOR 11	FACTOR 12	Communality prop of variance in the item attributatble to common factors)
**XSU038**	**.**	**.**	**.**	**.**	**.**	**.**	**.**	**.**	**.**	**.**	**.**	**.**	**0.26**
**XSU039**	**.**	**52**	**.**	**.**	**.**	**.**	**.**	**.**	**.**	**.**	**.**	**.**	**0.40**
**XSU040**	**.**	**60**	**.**	**.**	**.**	**.**	**.**	**.**	**.**	**.**	**.**	**.**	**0.50**
**XSU041**	**.**	**65**	**.**	**.**	**.**	**.**	**.**	**.**	**.**	**.**	**.**	**.**	**0.43**
**XSU042**	**.**	**.**	**.**	**.**	**.**	**.**	**.**	**.**	**.**	**.**	**.**	**.**	**0.33**
**XSU043**	**.**	**71**	**.**	**.**	**.**	**.**	**.**	**.**	**.**	**.**	**.**	**.**	**0.59**
**XSU044**	**.**	**76**	**.**	**.**	**.**	**.**	**.**	**.**	**.**	**.**	**.**	**.**	**0.64**
**XSU045**	**.**	**.**	**.**	**.**	**.**	**.**	**.**	**.**	**.**	**.**	**.**	**.**	**0.52**
**XSU046**	**.**	**.**	**.**	**.**	**.**	**.**	**.**	**.**	**.**	**.**	**.**	**.**	**0.29**
**XSU047**	**.**	**.**	**.**	**.**	**.**	**.**	**.**	**.**	**.**	**.**	**.**	**.**	**0.27**
**XSU048**	**40**	**.**	**.**	**.**	**.**	**.**	**.**	**.**	**.**	**.**	**.**	**.**	**0.35**
**XSU051**	**45**	**.**	**.**	**.**	**.**	**.**	**.**	**.**	**.**	**.**	**.**	**.**	**0.48**
**XSU052**	**.**	**.**	**.**	**.**	**.**	**.**	**.**	**.**	**.**	**.**	**.**	**.**	**0.29**
**XSU053**	**74**	**.**	**.**	**.**	**.**	**.**	**.**	**.**	**.**	**.**	**.**	**.**	**0.62**
**XSU054**	**76**	**.**	**.**	**.**	**.**	**.**	**.**	**.**	**.**	**.**	**.**	**.**	**0.69**
**XSU055**	**72**	**.**	**.**	**.**	**.**	**.**	**.**	**.**	**.**	**.**	**.**	**.**	**0.64**
**XSU056**	**60**	**.**	**.**	**.**	**.**	**.**	**.**	**.**	**.**	**.**	**.**	**.**	**0.42**
**XSU049**	**.**	**.**	**.**	**.**	**97**	**.**	**.**	**.**	**.**	**.**	**.**	**.**	**0.89**
**XSU050**	**.**	**.**	**.**	**.**	**94**	**.**	**.**	**.**	**.**	**.**	**.**	**.**	**0.85**
**XSU057**	**.**	**.**	**.**	**.**	**.**	**.**	**.**	**.**	**.**	**.**	**.**	**65**	**0.43**
**XSU058**	**.**	**.**	**.**	**.**	**.**	**.**	**.**	**.**	**.**	**.**	**.**	**80**	**0.63**
**XSU059**	**.**	**.**	**.**	**.**	**.**	**.**	**.**	**.**	**.**	**.**	**.**	**.**	**0.21**
**XSU065**	**.**	**.**	**.**	**.**	**.**	**.**	**.**	**.**	**.**	**.**	**.**	**.**	**0.19**
**XSU066**	**.**	**.**	**.**	**81**	**.**	**.**	**.**	**.**	**.**	**.**	**.**	**.**	**0.65**
**XSU067**	**.**	**.**	**.**	**77**	**.**	**.**	**.**	**.**	**.**	**.**	**.**	**.**	**0.66**
**XSU068**	**.**	**.**	**.**	**70**	**.**	**.**	**.**	**.**	**.**	**.**	**.**	**.**	**0.54**
**XSU069**	**.**	**.**	**.**	**59**	**.**	**.**	**.**	**.**	**.**	**.**	**.**	**.**	**0.41**
**XSU070**	**.**	**.**	**.**	**.**	**.**	**.**	**.**	**.**	**.**	**.**	**.**	**.**	**0.26**
**XSU071**	**.**	**.**	**.**	**.**	**.**	**.**	**82**	**.**	**.**	**.**	**.**	**.**	**0.69**
**XSU072**	**.**	**.**	**.**	**.**	**.**	**.**	**97**	**.**	**.**	**.**	**.**	**.**	**0.92**
**XSU073**	**.**	**.**	**.**	**.**	**.**	**.**	**53**	**.**	**.**	**.**	**.**	**.**	**0.45**
**XSU074**	**.**	**.**	**.**	**.**	**.**	**61**	**.**	**.**	**.**	**.**	**.**	**.**	**0.49**
**XSU075**	**.**	**.**	**.**	**.**	**.**	**92**	**.**	**.**	**.**	**.**	**.**	**.**	**0.90**
**XSU076**	**.**	**.**	**.**	**.**	**.**	**87**	**.**	**.**	**.**	**.**	**.**	**.**	**0.84**
**XSU077**	**.**	**.**	**.**	**.**	**.**	**.**	**.**	**.**	**67**	**.**	**.**	**.**	**0.62**
**XSU078**	**.**	**.**	**.**	**.**	**.**	**.**	**.**	**.**	**59**	**.**	**.**	**.**	**0.59**
**XSU079**	**.**	**.**	**.**	**.**	**.**	**.**	**.**	**.**	**.**	**.**	**.**	**.**	**0.43**
**XSU080**	**.**	**.**	**.**	**.**	**.**	**.**	**.**	**.**	**47**	**.**	**.**	**.**	**0.35**
**XSU081**	**.**	**.**	**.**	**.**	**.**	**.**	**.**	**.**	**.**	**.**	**.**	**.**	**0.24**
**XSU082**	**.**	**.**	**.**	**.**	**.**	**.**	**.**	**.**	**.**	**.**	**.**	**.**	**0.10**
**XSU083**	**.**	**.**	**.**	**.**	**.**	**.**	**.**	**.**	**.**	**.**	**.**	**.**	**0.16**
**XSU084**	**.**	**.**	**.**	**.**	**.**	**.**	**.**	**.**	**.**	**.**	**.**	**.**	**0.35**
**XSU085**	**.**	**.**	**.**	**.**	**.**	**.**	**.**	**45**	**.**	**.**	**.**	**.**	**0.42**
**XSU086**	**.**	**.**	**.**	**.**	**.**	**.**	**.**	**84**	**.**	**.**	**.**	**.**	**0.75**
**XSU087**	**.**	**.**	**.**	**.**	**.**	**.**	**.**	**80**	**.**	**.**	**.**	**.**	**0.72**
**XSU088**	**.**	**.**	**79**	**.**	**.**	**.**	**.**	**.**	**.**	**.**	**.**	**.**	**0.58**
**XSU089**	**.**	**.**	**91**	**.**	**.**	**.**	**.**	**.**	**.**	**.**	**.**	**.**	**0.78**
**XSU090**	**.**	**.**	**76**	**.**	**.**	**.**	**.**	**.**	**.**	**.**	**.**	**.**	**0.57**
**XSU091**	**.**	**.**	**73**	**.**	**.**	**.**	**.**	**.**	**.**	**.**	**.**	**.**	**0.57**
**XSU092**	**.**	**.**	**.**	**.**	**.**	**.**	**.**	**.**	**.**	**52**	**.**	**.**	**0.31**
**XSU093**	**.**	**.**	**.**	**.**	**.**	**.**	**.**	**.**	**.**	**70**	**.**	**.**	**0.51**
**XSU094**	**.**	**.**	**.**	**.**	**.**	**.**	**.**	**.**	**.**	**49**	**.**	**.**	**0.32**
**XSU095**	**.**	**.**	**.**	**.**	**.**	**.**	**.**	**.**	**.**	**.**	**.**	**.**	**0.25**
**XSU099**	**.**	**.**	**.**	**.**	**.**	**.**	**.**	**.**	**.**	**.**	**.**	**.**	**0.30**
**XSU100**	**.**	**.**	**.**	**.**	**.**	**.**	**.**	**.**	**.**	**.**	**59**	**.**	**0.42**
**XSU101**	**.**	**.**	**.**	**.**	**.**	**.**	**.**	**.**	**.**	**.**	**60**	**.**	**0.39**
**XSU102**	**.**	**.**	**.**	**.**	**.**	**.**	**.**	**.**	**.**	**.**	**40**	**.**	**0.27**
**XSU103**	**.**	**.**	**.**	**.**	**.**	**.**	**.**	**.**	**.**	**.**	**.**	**.**	**0.07**

**Fig. 2:**
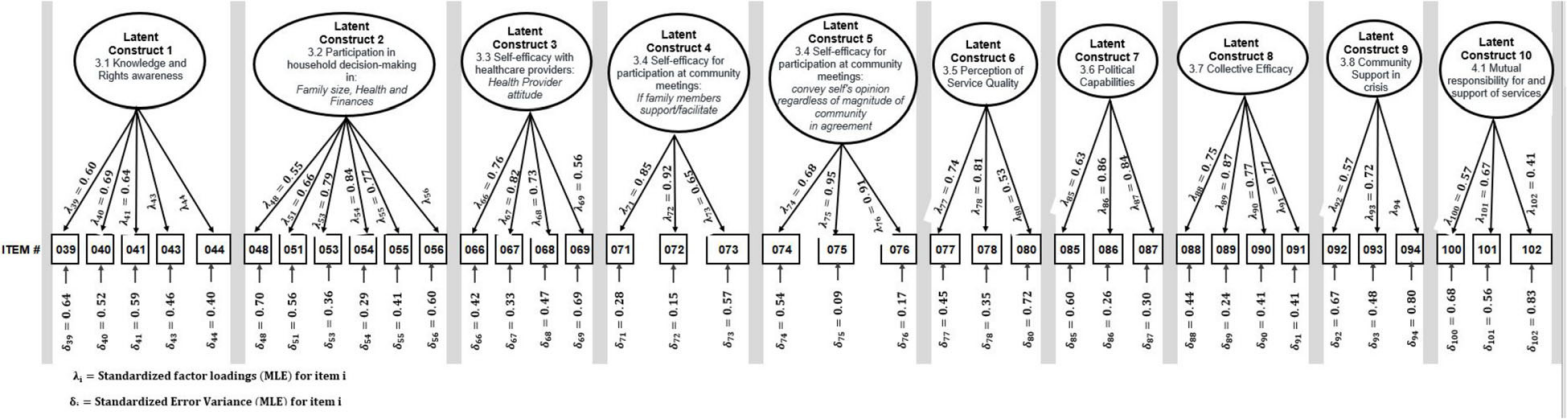
CFA structural model for Tanzania

To name the factors, we assessed what items were retained from the reliability analysis and the exploratory factor analysis and the original domain groupings. Factor 1 continues to reflect the domain items related to a client’s knowledge of their health rights and is named ‘Knowledge of health rights.’ The items included in factor 2 reflected knowledge of household decision-making in relation to finances and sexual and reproductive health and retained the name ‘Women’s participation in household decision-making’. The items included in factor 4 relate to clients’ perceived mistreatment by health care providers and is named ‘Mistreatment by health workers’. Items in factor 5 relate to the clients’ ability to attend a community meeting and are named ‘Ability to attend a community meeting.’ Factor 6 included items related to the ability to actively participate in a community meeting and is named ‘Ability to participate in a community meeting.’ Items in factor 7 relate to the client’s ‘Perception of quality’ and retain the name, ‘Perception of quality services’. The items in factor 8 relate to the understanding of how to bring about change in contraceptive clinics and are named ‘Awareness of accountability mechanisms.’ Factor 9 includes items that relate to the sense of social capital and cohesion and retains the name ‘Collective efficacy’. Factor 10 included items related to a sense of support from others during a crisis and retains the name ‘Community support in the time of crisis.’ For factor 11, the items related to how clients thought change could be achieved and retains the name ‘Mutual responsibility for and support of services’ (Fig. 2).

### Ghana

We started with a reliability analysis using the Cronbach’s alpha to determine item -to- item correlation of all 58 observed items and determined the overall alpha for each subscales in Ghana (see Table 8). The standardized alpha was greater than 0.60 for 8 of the 11 subscales and was retained. When the alpha was lesser than 0.60, items were removed if their removal improved the overall alpha scale. A total of 8 items were removed; this included questions 38, 39, 41, 65, 70, 82, 83, and 84. Scales with Cronbach’s alpha of ≥.60 were retained.

**Table 8:** Reliability Analysis in Ghana

**Qn #**	Scale mean if item deleted	Scale Standard Deviation	Variance	Item total Correlation with Total	Alpha if item deleted
Knowledge and Awareness of Rights					
38*	2.78	1.50	2.24	0.19	0.69
39	2.23	1.19	1.42	0.13	0.70
40	1.45	0.58	0.34	0.51	0.61
41	1.64	0.99	0.99	0.23	0.68
42	2.00	0.99	0.98	0.37	0.64
43	1.63	0.71	0.51	0.54	0.60
44	1.55	0.61	0.37	0.54	0.60
45	1.55	0.70	0.50	0.45	0.62
**Reliability Coeffcients**		Alpha = 0.74		N of cases 750	N of items: 5
Women’s participation in household decision-making (all items)					
46†	1.78	0.41	0.17	0.69	0.90
47†	1.75	0.44	0.19	0.69	0.90
48†	1.82	0.39	0.15	0.69	0.90
49†	1.81	0.39	0.15	0.69	0.90
50†	1.78	0.41	0.17	0.69	0.90
51†	1.82	0.38	0.15	0.69	0.90
52†	1.78	0.42	0.17	0.69	0.91
53†	1.87	0.33	0.11	0.69	0.91
54†	1.88	0.33	0.11	0.69	0.91
55†	1.88	0.33	0.11	0.69	0.91
56†	1.88	0.33	0.11	0.69	0.91
**Reliability Coeffcients**		Alpha = 0.91		N of cases 750	N of items: 10
Self efficacy with health care providers (Qn 57, 58 & 59)					
57**	0.89	0.31	0.10	0.50	0.65
58**	0.96	1.20	1.44	0.64	0.46
59**	0.93	0.26	0.07	0.44	0.72
**Reliability Coeffcients**		Alpha = 0.71		N of cases 750	N of items: 2
Self efficacy with health care providers (all items)					
65	1.82	0.98	0.96	−0.05	0.73
66*	2.13	1.14	1.29	0.48	0.54
67*	1.94	0.92	0.85	0.65	0.47
68*	1.95	0.89	0.80	0.54	0.52
69*	2.05	0.98	0.97	0.49	0.54
70	1.49	0.78	0.61	0.16	0.66
**Reliability Coeffcients**		Alpha = 0.80		N of cases 750	N of items: 4
Self-efficacy for participation at community meetings (all items)(Qn 71–73)					
71	2.44	1.65	2.72	0.73	0.85
72	2.60	1.63	2.66	0.80	0.83
73	2.60	1.62	2.62	0.60	0.91
**Reliability Coeffcients**		Alpha = 0.90		N of cases 750	N of items:2
Self-efficacy for participation at community meetings (all items)					
74	1.67	1.15	1.32	0.52	0.86
75	2.07	1.33	1.77	0.76	0.61
76	2.35	1.45	2.10	0.69	0.70
**Reliability Coeffcients**		Alpha = 0.81		N of cases 750	N of items: 2
Perception of service quality (all items)					
77	1.51	0.56	0.31	0.42	0.55
78	1.55	0.56	0.31	0.53	0.51
79	1.57	0.60	0.36	0.46	0.54
80	1.74	0.74	0.55	0.35	0.58
81	1.89	0.82	0.67	0.31	0.59
82	2.06	1.15	1.32	0.09	0.68
**Reliability Coeffcients**		Alpha = 0.68		N of cases 750	N of items: 5
Political capabilities					
83	1.76	0.79	0.62	0.14	0.53
84	2.33	1.17	1.37	0.11	0.56
85	2.02	0.99	0.98	0.38	0.39
86	2.79	1.24	1.54	0.40	0.39
87	2.40	1.20	1.44	0.44	0.35
**Reliability Coeffcients**		Alpha = 0.64		N of cases 750	N of items:3
**Collective efficacy (all items)**					
88	1.47	0.89	0.79	0.58	0.74
89	1.46	0.82	0.67	0.68	0.69
90	1.49	0.82	0.67	0.52	0.77
91	1.43	0.76	0.58	0.61	0.73
**Reliability Coeffcients**		Alpha = 0.79		N of cases 750	N of items:4
Community support in time of crisis (all items)					
92	1.25	0.71	0.50	0.42	0.62
93	1.28	0.70	0.49	0.50	0.57
94	1.57	1.11	1.23	0.48	0.58
95	1.87	1.35	1.82	0.40	0.63
**Reliability Coeffcients**		Alpha = 0.67		N of cases 750	N of items:3
Mutual responsibility for and support of services (all items)					
99¥	1.69	0.47	0.22	0.58	0.83
100¥	1.35	0.75	0.56	0.58	0.83
101¥	1.54	0.51	0.26	0.71	0.79
102¥	1.54	0.51	0.26	0.73	0.78
103¥	1.41	0.50	0.25	0.64	0.81
**Reliability Coeffcients**		Alpha = 0.84		N of cases 750	N of items:4

* Reverse coded

** Yes/No Response items

† 2 Response items

¥ 3 Response items

In the KMO measure of sampling adequacy, all items had a KMO of 0.5, and the overall KMO score was 0.80, suggesting that there is sufficient correlation between the variables to conduct exploratory factor analysis.

The EFA used principal factors with oblique oblimin rotation yielded 13 factors with eigenvalues greater than or equal to 1.0 (see Table 9).

**Table 9:** Eigenvalues in Ghana

Factor	Eigenvalue
**1**	18.47
**2**	13.98
**3**	11.17
**4**	7.68
**5**	6.27
**6**	5.43
**7**	4.18
**8**	3.78
**9**	2.60
**10**	2.32
**11**	1.99
**12**	1.45
**13**	1.31

The revised item data was used in the confirmatory factor analysis to analyze a good fit. The SRMR score of 0.05, the CFI score of 0.94, and the RMSEA score of 0.03 are good. All three score suggests acceptable goodness-of-fit.

In total, seven items loaded on factor 1, five items loaded on factor 2, three items loaded on to factor 3, four items loaded on to factor 4, four items loaded on to factor 5, five items loaded onto factor 6, three items loaded onto factor 7, three items loaded onto factor 8, three items loaded onto factor 9, four items loaded onto factor 10, four items loaded onto factor 11, two items on factor 12 and one item on factor 13 Table 10. Factors with less than two items were excluded as this is not sufficient for factor analysis, this reduced the number of factors from 13 to 10. We discarded items with factor loadings less than 0.4, and two items were removed, see Table 11. In total, 10 items were removed, and 48 items were retained.

**Table 10:** Factor loadings for Ghana

XSU QN #	FACTOR 1	FACTOR 2	FACTOR 3	FACTOR 4	FACTOR 5	FACTOR 6	FACTOR 7	FACTOR 8	FACTOR 9	FACTOR 10	FACTOR 11	FACTOR 12	FACTOR 13	Communality (prop of variance in the item attributatble to common factors)
**XSU038**	**.**	**.**	**.**	**.**	**.**	**.**	**.**	**.**	**.**	**.**	**.**	**.**	**.**	**0.36**
**XSU039**	**.**	**.**	**.**	**.**	**.**	**.**	**.**	**.**	**.**	**.**	**.**	**.**	**.**	**0.25**
**XSU040**	**.**	**.**	**.**	**.**	**.**	**41**	**.**	**.**	**.**	**.**	**.**	**.**	**.**	**0.33**
**XSU041**	**.**	**.**	**.**	**.**	**.**	**.**	**.**	**.**	**.**	**.**	**.**	**.**	**.**	**0.11**
**XSU042**	**.**	**.**	**.**	**.**	**.**	**52**	**.**	**.**	**.**	**.**	**.**	**.**	**.**	**0.29**
**XSU043**	**.**	**.**	**.**	**.**	**.**	**71**	**.**	**.**	**.**	**.**	**.**	**.**	**.**	**0.50**
**XSU044**	**.**	**.**	**.**	**.**	**.**	**65**	**.**	**.**	**.**	**.**	**.**	**.**	**.**	**0.46**
**XSU045**	**.**	**.**	**.**	**.**	**.**	**47**	**.**	**.**	**.**	**.**	**.**	**.**	**.**	**0.29**
**XSU046**	**59**	**.**	**.**	**.**	**.**	**.**	**.**	**.**	**.**	**.**	**.**	**.**	**.**	**0.54**
**XSU047**	**70**	**.**	**.**	**.**	**.**	**.**	**.**	**.**	**.**	**.**	**.**	**.**	**.**	**0.57**
**XSU048**	**78**	**.**	**.**	**.**	**.**	**.**	**.**	**.**	**.**	**.**	**.**	**.**	**.**	**0.64**
**XSU051**	**90**	**.**	**.**	**.**	**.**	**.**	**.**	**.**	**.**	**.**	**.**	**.**	**.**	**0.73**
**XSU052**	**80**	**.**	**.**	**.**	**.**	**.**	**.**	**.**	**.**	**.**	**.**	**.**	**.**	**0.65**
**XSU053**	**63**	**.**	**.**	**.**	**.**	**.**	**.**	**.**	**.**	**.**	**.**	**.**	**.**	**0.54**
**XSU054**	**56**	**.**	**.**	**.**	**.**	**.**	**.**	**.**	**.**	**.**	**.**	**.**	**.**	**0.43**
**XSU055**	**.**	**.**	**.**	**.**	**.**	**.**	**.**	**.**	**.**	**.**	**.**	**64**	**.**	**0.66**
**XSU056**	**.**	**.**	**.**	**.**	**.**	**.**	**.**	**.**	**.**	**.**	**.**	**69**	**.**	**0.68**
**XSU049**	**.**	**.**	**.**	**.**	**.**	**.**	**.**	**.**	**.**	**.**	**.**	**.**	**.**	**0.39**
**XSU050**	**43**	**.**	**.**	**.**	**.**	**.**	**.**	**.**	**.**	**.**	**.**	**.**	**.**	**0.41**
**XSU051**	**.**	**.**	**.**	**.**	**.**	**.**	**.**	**73**	**.**	**.**	**.**	**.**	**.**	**0.63**
**XSU058**	**.**	**.**	**.**	**.**	**.**	**.**	**.**	**82**	**.**	**.**	**.**	**.**	**.**	**0.66**
**XSU059**	**.**	**.**	**.**	**.**	**.**	**.**	**.**	**58**	**.**	**.**	**.**	**.**	**.**	**0.35**
**XSU065**	**.**	**.**	**.**	**.**	**.**	**.**	**.**	**.**	**.**	**.**	**.**	**.**	**.**	**0.13**
**XSU066**	**.**	**.**	**.**	**70**	**.**	**.**	**.**	**.**	**.**	**.**	**.**	**.**	**.**	**0.52**
**XSU067**	**.**	**.**	**.**	**84**	**.**	**.**	**.**	**.**	**.**	**.**	**.**	**.**	**.**	**0.71**
**XSU068**	**.**	**.**	**.**	**70**	**.**	**.**	**.**	**.**	**.**	**.**	**.**	**.**	**.**	**0.50**
**XSU069**	**.**	**.**	**.**	**60**	**.**	**.**	**.**	**.**	**.**	**.**	**.**	**.**	**.**	**0.43**
**XSU070**	**.**	**.**	**.**	**.**	**.**	**.**	**.**	**.**	**.**	**.**	**.**	**.**	**.**	**0.20**
**XSU071**	**.**	**.**	**90**	**.**	**.**	**.**	**.**	**.**	**.**	**.**	**.**	**.**	**.**	**0.83**
**XSU072**	**.**	**.**	**91**	**.**	**.**	**.**	**.**	**.**	**.**	**.**	**.**	**.**	**.**	**0.87**
**XSU073**	**.**	**.**	**72**	**.**	**.**	**.**	**.**	**.**	**.**	**.**	**.**	**.**	**.**	**0.65**
**XSU074**	**.**	**.**	**.**	**.**	**.**	**.**	**58**	**.**	**.**	**.**	**.**	**.**	**.**	**0.43**
**XSU075**	**.**	**.**	**.**	**.**	**.**	**.**	**97**	**.**	**.**	**.**	**.**	**.**	**.**	**0.89**
**XSU076**	**.**	**.**	**.**	**.**	**.**	**.**	**76**	**.**	**.**	**.**	**.**	**.**	**.**	**0.68**
**XSU077**	**.**	**.**	**.**	**.**	**.**	**.**	**.**	**.**	**.**	**57**	**.**	**.**	**.**	**0.39**
**XSU078**	**.**	**.**	**.**	**.**	**.**	**.**	**.**	**.**	**.**	**73**	**.**	**.**	**.**	**0.60**
**XSU079**	**.**	**.**	**.**	**.**	**.**	**.**	**.**	**.**	**.**	**60**	**.**	**.**	**.**	**0.40**
**XSU080**	**.**	**.**	**.**	**.**	**.**	**.**	**.**	**.**	**.**	**.**	**.**	**.**	**49**	**0.42**
**XSU081**	**.**	**.**	**.**	**.**	**.**	**.**	**.**	**.**	**.**	**.**	**.**	**.**	**.**	**0.22**
**XSU082**	**.**	**.**	**.**	**.**	**.**	**.**	**.**	**.**	**.**	**.**	**.**	**.**	**.**	**0.24**
**XSU083**	**.**	**.**	**.**	**.**	**.**	**.**	**.**	**.**	**.**	**.**	**.**	**.**	**.**	**0.16**
**XSU084**	**.**	**.**	**.**	**.**	**.**	**.**	**.**	**.**	**.**	**.**	**.**	**.**	**.**	**0.33**
**XSU085**	**.**	**.**	**.**	**.**	**.**	**.**	**.**	**.**	**61**	**.**	**.**	**.**	**.**	**0.62**
**XSU086**	**.**	**.**	**.**	**.**	**.**	**.**	**.**	**.**	**65**	**.**	**.**	**.**	**.**	**0.65**
**XSU087**	**.**	**.**	**.**	**.**	**.**	**.**	**.**	**.**	**67**	**.**	**.**	**.**	**.**	**0.62**
**XSU088**	**.**	**.**	**.**	**.**	**73**	**.**	**.**	**.**	**.**	**.**	**.**	**.**	**.**	**0.58**
**XSU089**	**.**	**.**	**.**	**.**	**85**	**.**	**.**	**.**	**.**	**.**	**.**	**.**	**.**	**0.73**
**XSU090**	**.**	**.**	**.**	**.**	**56**	**.**	**.**	**.**	**.**	**.**	**.**	**.**	**.**	**0.35**
**XSU091**	**.**	**.**	**.**	**.**	**64**	**.**	**.**	**.**	**.**	**.**	**.**	**.**	**.**	**0.42**
**XSU092**	**.**	**.**	**.**	**.**	**.**	**.**	**.**	**.**	**.**	**.**	**60**	**.**	**.**	**0.39**
**XSU093**	**.**	**.**	**.**	**.**	**.**	**.**	**.**	**.**	**.**	**.**	**72**	**.**	**.**	**0.50**
**XSU094**	**.**	**.**	**.**	**.**	**.**	**.**	**.**	**.**	**.**	**.**	**52**	**.**	**.**	**0.35**
**XSU095**	**.**	**.**	**.**	**.**	**.**	**.**	**.**	**.**	**.**	**.**	**43**	**.**	**.**	**0.34**
**XSU099**	**.**	**64**	**.**	**.**	**.**	**.**	**.**	**.**	**.**	**.**	**.**	**.**	**.**	**0.43**
**XSU100**	**.**	**61**	**.**	**.**	**.**	**.**	**.**	**.**	**.**	**.**	**.**	**.**	**.**	**0.52**
**XSU101**	**.**	**81**	**.**	**.**	**.**	**.**	**.**	**.**	**.**	**.**	**.**	**.**	**.**	**0.67**
**XSU102**	**.**	**82**	**.**	**.**	**.**	**.**	**.**	**.**	**.**	**.**	**.**	**.**	**.**	**0.76**
**XSU103**	**.**	**65**	**.**	**.**	**.**	**.**	**.**	**.**	**.**	**.**	**.**	**.**	**.**	**0.56**

**Table 11:** Final items per country

Tanzania				Ghana			
No	Item	CFA Factor loading	Standard Error Variance	No	Item	CFA Factor loading	Standard Error Variance
**Knowledge of Health Rights**							
**XSU038**	A healthcare provider can refuse to provide me family planning services because of who I am. (reverse-coded)			**XSU038**	A healthcare provider can refuse to provide me family planning services because of who I am. (reverse-coded)		
**XSU039**	The government ensures that family planning methods (Ghana) or services (Tanzania) are free of cost.	0.6	0.64	**XSU039**	The government ensures that family planning methods (Ghana) or services (Tanzania) are free of cost.		
**XSU040**	I have the right to privacy during my family planning visit.	0.69	0.52	**XSU040**	I have the right to privacy during my family planning visit.	0.53	0.72
**XSU041**	The healthcare provider should not share my information with other people.	0.64	0.46	**XSU041**	The healthcare provider should not share my information with other people.	0.44	0.8
**XSU042**	If I am unhappy with the care I received, I know there are ways to make a complaint.			**XSU042**	If I am unhappy with the care I received, I know there are ways to make a complaint.	0.7	0.5
**XSU043**	Healthcare providers must answer all my health related questions.	0.73	0.46	**XSU043**	Healthcare providers must answer all my health related questions.	0.7	0.51
**XSU044**	Healthcare providers should inform me about the different family planning options.	0.77	0.4	**XSU044**	Healthcare providers should inform me about the different family planning options.	0.54	0.71
**XSU045**	I can refuse any family planning method offered if I do not want to use it.			**XSU045**	I can refuse any family planning method offered if I do not want to use it.		
**Mistreatment by Health workers**							
**XSU065**	I felt like I could discuss my problems, question and concerns with the health care provider without feeling embarrassed.			**XSU065**	I felt like I could discuss my problems, question and concerns with the health care provider without feeling embarrassed.		
**XSU066**	One of the providers or staff refused to offer me the service I wanted to receive. (reverse-coded)	0.76	0.42	**XSU066**	One of the providers or staff refused to offer me the service I wanted to receive. (reverse-coded)	0.71	0.5
**XSU067**	The provider ignored my request or my preferences today.	0.82	0.33	**XSU067**	The provider ignored my request or my preferences today.	0.86	0.26
**XSU068**	I felt like the provider did not listen to what I was saying (reverse-coded)	0.73	0.47	**XSU068**	I felt like the provider did not listen to what I was saying (reverse-coded)	0.66	0.56
**XSU069**	A provider strongly encouraged me to use one family planning that was different to the one I wanted (reverse-coded)	0.56	0.69	**XSU069**	A provider strongly encouraged me to use one family planning that was different to the one I wanted (reverse-coded)	0.6	0.64
**XSU070**	I have the right to choose my family planning method.			**XSU070**	I have the right to choose my family planning method.		
**Perception of quality services**							
**XSU077**	The staff at this health facility have high quality family planning services.	0.74	0.45	**XSU077**	The staff at this health facility have high quality family planning services.	0.62	0.61
**XSU078**	The staff at this health facility ensures privacy and confidentiality when providing services.	0.81	0.35	**XSU078**	The staff at this health facility ensures privacy and confidentiality when providing services.	0.81	0.35
**XSU079**	The health facility is clean.	0.53	0.73	**XSU079**	The health facility is clean.	0.57	0.68
**XSU080**	At this health facility, if women choose, they can bring their husband/partner for the family planning consultation.			**XSU080**	At this health facility, if women choose, they can bring their husband/partner for the family planning consultation.		
**XSU081**	At this health facility, if women choose, they can bring a family member or friend for the family planning consultation.			**XSU081**	At this health facility, if women choose, they can bring a family member or friend for the family planning consultation.		
**XSU082**	Unmarried women can access family planning and reproductive health service at the health facility.			**XSU082**	Unmarried women can access family planning and reproductive health service at the health facility.		
**Women’s participation in h/h decision-making**							
**XSU046**	First, would you tell me which member of your household usually makes decisions about your health care?			**XSU046**	First, would you tell me which member of your household usually makes decisions about your health care?		0.49
**XSU047**	Which member of your household usually makes decisions about making large household purchases?			**XSU047**	Which member of your household usually makes decisions about making large household purchases?	0.75	0.44
**XSU048**	Which member of your household usually makes decisions about making household purchases for daily needs?	0.55	0.7	**XSU048**	Which member of your household usually makes decisions about making household purchases for daily needs?	0.8	0.36
**XSU049**	Which member of your household usually makes decisions about when you will visit family/relatives/friends?			**XSU049**	Which member of your household usually makes decisions about when you will visit family/relatives/friends?	0.83	0.32
**XSU050**	Which member of your household usually makes decisions about when your whole household will visit family/relatives/friends?			**XSU050**	Which member of your household usually makes decisions about when your whole household will visit family/relatives/friends?	0.74	0.45
**XSU051**	Which member of your household usually makes decisions about how to use the money that you bring into the household?	0.66	0.56	**XSU051**	Which member of your household usually makes decisions about how to use the money that you bring into the household?	0.73	0.47
**XSU052**	Which member of your household usually makes decisions about how to use the money your husband/partner brings into the household?			**XSU052**	Which member of your household usually makes decisions about how to use the money your husband/partner brings into the household?	0.64	0.59
**XSU053**	Which member of your household usually makes decisions about whether you or you and your husband/partner use family planning?	0.79	0.36	**XSU053**	Which member of your household usually makes decisions about whether you or you and your husband/partner use family planning?		
**XSU054**	Which member of your household usually makes decisions about where you will receive family planning?	0.84	0.29	**XSU054**	Which member of your household usually makes decisions about where you will receive family planning?		
**XSU055**	Which member of your household usually makes decisions about if you will be tested for the AIDS virus?	0.77	0.41	**XSU055**	Which member of your household usually makes decisions about if you will be tested for the AIDS virus?		
**XSU056**	Which member of your household usually makes decisions about how many children you will have?	0.63	0.6	**XSU056**	Which member of your household usually makes decisions about how many children you will have?	0.59	0.66
**Self efficacy with health care providers**							
**XSU057**	After your consultation with the health care provider today, do you know what your reproductive and family planning options are?			**XSU057**	After your consultation with the health care provider today, do you know what your reproductive and family planning options are?	0.73	0.47
**XSU058**	After your consultation with the health care provider today, do you feel that you can act on your choice for family planning?			**XSU058**	After your consultation with the health care provider today, do you feel that you can act on your choice for family planning?	0.87	0.24
**XSU059**	Do you know what help you need to make a decision?			**XSU059**	Do you know what help you need to make a decision?	0.54	0.71
**Ability to atttend community meetings**							
**XSU071**	How sure are you that you could attend a community meeting if your family did not support you to participate?	0.85	0.28	**XSU071**	How sure are you that you could attend a community meeting if your family did not support you to participate?	0.88	0.21
**XSU072**	How sure are you that you could attend a community meeting if your family said you could not go?	0.92	0.15	**XSU072**	How sure are you that you could attend a community meeting if your family said you could not go?	0.93	0.14
**XSU073**	How sure are you that you could attend a community meeting if your family would not help with your household duties so that you could attend?	0.65	0.57	**XSU073**	How sure are you that you could attend a community meeting if your family would not help with your household duties so that you could attend?	0.79	0.38
**Ability to participate in community meetings**							
**XSU074**	How sure are you that you could express your opinion at a community meeting?	0.68	0.54	**XSU074**	How sure are you that you could express your opinion at a community meeting?	0.58	0.66
**XSU075**	How sure are you that you could express your opinion at a community meeting if a few people did not agree with what you were saying?	0.95	0.09	**XSU075**	How sure are you that you could express your opinion at a community meeting if a few people did not agree with what you were saying?	0.9	0.18
**XSU076**	How sure are you that you could express your opinion at a community meeting if many people did not agree with what you were saying?	0.91	0.17	**XSU076**	How sure are you that you could express your opinion at a community meeting if many people did not agree with what you were saying?	0.82	0.33
**Awareness of accountabilty mechanisms**							
**XSU083**	Today, if I went to the clinic I believe I could get family planning I wanted without facing any barriers of cost, age and marital status.			**XSU083**	Today, if I went to the clinic I believe I could get family planning I wanted without facing any barriers of cost, age and marital status.		
**XSU084**	Anyone outside of the clinic, like friends or community members, can help you access your right to quality family planning services.			**XSU084**	Anyone outside of the clinic, like friends or community members, can help you access your right to quality family planning services.		
**XSU085**	Health providers and district government officials can directly influence the quality of your local family planning services?	0.63	0.6	**XSU085**	Health providers and district government officials can directly influence the quality of your local family planning services?	0.52	0.73
**XSU086**	Challenging people of influence is the best way to change family planning services in the clinic.	0.86	0.26	**XSU086**	Challenging people of influence is the best way to change family planning services in the clinic.	0.48	0.77
**XSU087**	Collaboration with people of influence is the best way to change family planning services in the clinic.	0.84	0.84	**XSU087**	Collaboration with people of influence is the best way to change family planning services in the clinic.	0.96	0.08
**Mutual responsibility for and support of services (all items)**							
**XSU099**	Who could have the most impact on making sure that women are treated with respect by health workers?			**XSU099**	Who could have the most impact on making sure that women are treated with respect by health workers?	0.6	0.64
**XSU100**	Who could have the most impact on making sure that women have transportation to the hospital for permanent methods of contraception?	0.57	0.68	**XSU100**	Who could have the most impact on making sure that women have transportation to the hospital for permanent methods of contraception?	0.6	0.64
**XSU101**	Who could have the most impact on increasing the number of days a health worker visits your community?	0.67	0.56	**XSU101**	Who could have the most impact on increasing the number of days a health worker visits your community?	0.8	0.36
**XSU102**	Who could have the most impact on making sure the poorest and most vulnerable women in the community receive care?	0.41	0.83	**XSU102**	Who could have the most impact on making sure the poorest and most vulnerable women in the community receive care?	0.87	0.24
**XSU103**	Who could have the most impact on getting funding to improve health services in this community?			**XSU103**	Who could have the most impact on getting funding to improve health services in this community?	0.71	0.46
**Collective efficacy**							
**XSU088**	How sure are you that the people in your community could work together to improve family planning services in this community?	0.75	0.44	**XSU088**	How sure are you that the people in your community could work together to improve family planning services in this community?	0.75	0.44
**XSU089**	How sure are you that the people in your community could work together to improve how women are treated at the health facility?	0.87	0.24	**XSU089**	How sure are you that the people in your community could work together to improve how women are treated at the health facility?	0.85	0.28
**XSU090**	How sure are you that the people in your community could work together to obtain government services and entitlements?	0.77	0.41	**XSU090**	How sure are you that the people in your community could work together to obtain government services and entitlements?	0.55	0.7
**XSU091**	How sure are you that the people in your community could work together to improve the health and well-being of women in this community?	0.77	0.41	**XSU091**	How sure are you that the people in your community could work together to improve the health and well-being of women in this community?	0.63	0.61
**Community Support in the time of crisis**							
**XSU092**	How sure are you that there is someone in your community, apart from your immediate family, who you could go to for advice?			**XSU092**	How sure are you that there is someone in your community, apart from your immediate family, who you could go to for advice?	0.6	0.64
**XSU093**	How sure are you that there is someone in your community, apart from your immediate family, who could take you to the clinic?	0.57	0.67	**XSU093**	How sure are you that there is someone in your community, apart from your immediate family, who could take you to the clinic?	0.66	0.57
**XSU094**	How sure are you that there is someone in your community, apart from your immediate family, who would help care for your children or household while you are away?	0.72	0.48	**XSU094**	How sure are you that there is someone in your community, apart from your immediate family, who would help care for your children or household while you are away?	0.56	0.69
**XSU095**	How sure are you that there is someone in your community, apart from your immediate family, who would loan you money for transport?	0.44	0.8	**XSU095**	How sure are you that there is someone in your community, apart from your immediate family, who would loan you money for transport?	0.51	0.74

**Fig. 3:**
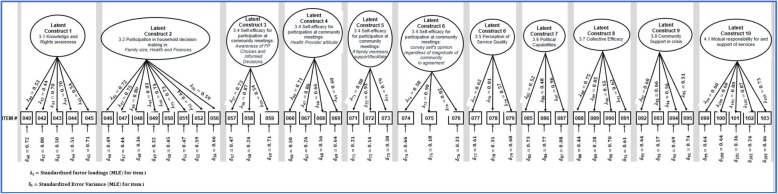
CFA structural model for Ghana

Similar to Tanzania, to name the factors, we assessed what items were retained and the original domains. These corresponded with those found in Tanzania, with the exception of one factor that was dropped in Tanzania but retained in Ghana. The items included in this factor related to a clients’ sense of activation and are named ‘patient activation.’ There was also a difference in what items were retained in relation to household decision making; those pertaining to contraception were excluded, whereas those related to decisions over finances, over seeking health care, and visiting others were included (Fig. 3).

The revised item data was used in the confirmatory factor analysis to assess the goodness-of-fit. The SRMR score of 0.057, the CFI score of 0.88, and the RMSEA score of 0.04, all suggest an acceptable goodness-of-fit.

## Discussion

We adapted and validated the measures of service users’ attitudes and behaviors in a social accountability process to improve contraceptive services in Ghana and Tanzania. The measure has high construct validity and reliability in both countries. We identified several subscales in both countries: 10 subscales in Tanzania and 11 subscales in Ghana. Many of the scales and items were shared across both settings, as shown in Table 11. There were some differences in how the scales performed in the different contexts; however, there was convergence in the analysis to suggest that this measure may be relevant to other settings.

The first group of scales related to clients’ awareness about their health and contraceptive entitlements. This includes the following scales: knowledge of health rights, mistreatment by health workers, and perception of quality. While these scales are conceptually distinct constructs, when combined, they measure a critical aspect of clients’ knowledge of their rights and entitlements. In the social accountability canon, being aware of one’s health rights and entitlements is a critical precursor to generating critical consciousness and people’s ability to participate in collective action [9, 12, 14, 29]. As George [12] reminds us, “Not only is access to information essential for improving health awareness and access, it is impossible to mobilise for change without it. People cannot demand services and accountability if they do not know what they need and what they are entitled to.”

Several of the scales related to capturing an individual’s perception of their ability to affect change are included: ‘Women’s participation in household decision-making’, ‘Self-efficacy with health care providers’, ‘Ability to attend community meetings’, and ‘Ability to participate in community meetings’. There was also a group of scales related to if clients knew how to improve their existing circumstance and included: ‘Mutual responsibility for and support of services’ and ‘Awareness of accountability mechanisms’. Many have scholars working in social accountability have argued that it is important the people see that change is possible, and they themselves are agents of change [9, 33].

The final group of scales were related to collective identity and action, and included: ‘Collective efficacy’ and ‘Community support in the time of crisis’. In collective action, social cohesion and social capital are central to the change process. This starts with groups of people initially identifying commonalities with each other moves towards a belief that the group can work together to bring about changes [11, 30, 31]. Such solidarities provided people with a sense of agency and collective identity necessary to confront unequal power [12, 14, 29].

The measures also speak to another gap in understanding the poor quality of care still reported in contraceptive services [1, 15, 37]. Harris et al. [15] argue that current tools do not adequately determine the prevalence or impact of negative client experiences in contraceptive programs and that current measures can de-emphasise and misdirect attention from client experiences of coercion, corruption, and disrespect and abuse when they come for family planning. The scale, ‘Mistreatment by health workers’, responds to this gap and by better capturing all dimensions of patients’ experience, we can learn what is working, or not, in terms of quality of care [2].

The study benefited from the rigorous methodology for the validation of psychometric scales [6, 27]. A limitation at this stage is that the test-retest was not conducted to examine if the measurement tools reliably replicate the result in the same situation and population. A test-retest was added to the end line data collection.

## Conclusion

In this paper, we share the findings from testing a 58-item scale to measure intermediate changes among health service users during a social accountability process to improve contraceptive services. The study suggests that the multi-dimensional scales have high construct validity and reliability in both countries. Though there were differences between contexts and in some of the items and scales, there was convergence in the analysis that suggests that this measure may be relevant to multiple settings and needs to be validated in new settings.

The refined tool resulting from the CaPSAI Project has both research and programmatic utility. It will be useful for research to understand the monitoring and evaluation of social accountability processes and could help develop and target interventions. The validated scales allow for a more robust measurement of the intermediate outcomes. This scale will facilitate measurement to improve community engagement in contraceptive programs.

## Data Availability

The data that support the findings of this study are available from the corresponding author upon reasonable request.

## Abbreviations

CaPSAI: Community and Provider Social Accountability Intervention
CFA: Confirmatory Factor Analysis
CFI: Comparative Fit Index
EFA: Exploratory Factor Analysis
KMO: Kaiser-Meyer-Olkin of Sampling Adequacy
MLE: Maximum Likelihood Estimation
NHS: National Health Service
RMSEA: Root Mean Square Error of Approximation
SRMR: Standardized Root Mean Square Residual
WHO: World Health Organization
